# A Multistep Iter for Functional Reconstruction in Mangled Upper Limb: A Retrospective Analysis of Integrated Surgical and Medical Approach

**DOI:** 10.3390/medicina56080398

**Published:** 2020-08-07

**Authors:** Francesco De Francesco, Andrea Marchesini, Andrea Campodonico, Alexander Dietrich Neuendorf, Pier Paolo Pangrazi, Michele Riccio

**Affiliations:** Department of General and Specialties Surgery, SOD of Reconstructive Surgery and Hand Surgery, AOU “OspedaliRiuniti”, 60126 Ancona, Italy; andrea.marchesini@ospedaliriuniti.marche.it (A.M.); andrea.campodonico@ospedaliriuniti.marche.it (A.C.); alexanderdietrich.neuendorf@ospedaliriuniti.marche.it (A.D.N.); pierpaolo.pangrazi@ospedaliriuniti.marche.it (P.P.P.); michele.riccio@ospedaliriuniti.marche.it (M.R.)

**Keywords:** mangled limb, post-traumatic wounds, contaminated wound, HBOT, NPT

## Abstract

*Background and objectives:* Complex limb wounds with multiple tissue involvement are commonly due to high energy trauma. Tissue damage is a dynamic entity and the exact extent of the injury is rarely instantly perceptible. Hence, reconstruction frequently involves a multi-stage procedure concluding with tissue replacement. *Materials and Methods:* A retrospective study was conducted between 2006 and 2018 and included 179 patients with contaminated multi-tissue injuries treated with hyperbaric oxygen therapy, negative pressure therapy, physiotherapy and drug treatment associated with multiple surgical time in a multistep approach, focusing on pain levels and wound closure rates. *Results:* Despite the long-term response to traumatic events, a combined approach of delayed surgical reconstructive time in mangled upper limb yielded satisfactory functional outcomes. *Conclusions:* The complex upper limb wound with deep tissue exposure may be treated with a multi-stage procedure alternatively to immediate reconstruction. The integrated technique enables the preservation of existing healthy tissue and concurrent radical debridement, reducing the risk of infection, as well as avoiding the loss of free flaps and dehiscence due to incorrect wound estimation.

## 1. Introduction

The incidence of complex trauma has decreased considerably over the years due to increasingly effective protection systems, public awareness campaigns and precise legislation regarding accident prevention on the workplace and elsewhere. Unfortunately, the reduced frequency of complex traumas is in parallel with an increase in severity due to the high kinetic energy of the trauma or deterioration of the unfavorable circumstances leading to the trauma.

A mangled injury is the result of multiple tissue damage which may involve the bone, skin, vasculature and the nervous system, provoking significant bone and soft-tissue loss and a high potential for contamination. [1]. These types of injuries are clinically challenging in terms of wound management due to the devastating consequences of the injury in itself—compression, crushing, heating and avulsion may induce deep tissue damage which is not immediately visible in emergency surgery. A complex reconstructive strategy is required to obtain effective functional outcomes and represents a challenge to both patients and surgeons. Healing wound failure is attributable to patient-related factors, infection, dehiscence or compromised immunity [2,3], which are relevant elements to outline when considering the potential benefits of a reconstructive approach. The lower limb area has been debated at length and exhaustively classified compared to the upper limb [4,5,6,7]. From a psychological point of view, the lower limb extremity is easier to manage—a foot amputation is considered less traumatic than a hand amputation [8,9]. A non-functional hand may undoubtedly be replaced by a prosthesis to address an authentic social function. Nevertheless, different approaches have been proposed for open wound and savable limb management. Immediate reconstruction with emergency free flaps [10,11,12] is less and less frequent when considering the potential waste of flaps and the event of dynamic wounds with progressive evolution and the likelihood of deterioration. Conversely, continuous wound dressings and plaster casts may produce chronic drainage and extended disablement as well as a ≥50% higher risk of infection, non-union of the bone and truncated limbs [13]. Combined treatment may be an appropriate approach: a complex trauma duly requires a complex treatment [14,15,16,17] with a “partially-delayed” and multi-step strategy. Negative pressure wound therapy has been used since the 1990s. Torbrand et al. [18] observed that negative pressure therapy (NPT) prompted the development of granulation tissue and wound edge contraction in swine models. Furthermore, NPT is frequently used for wound treatments and has been reported to decrease edema, increase skin perfusion and enhance granulation tissue formation. The mechanism of action includes a pump connected to a dressing in the form of a wound conduit with packing foam or gauze. The pump produces a suction pressure on the interface of the wound bed with a negative pressure range of 50–125 mmHg. According to Krug et al. in 2011, approximately 1000 peer-reviewed papers have investigated chronic wounds and the effectiveness and safety of NPT. [19]. In 2007, Kanakaris et al. also investigated the adequacy of NPT to manage wounds following lower extremity traumas or burns [20].

Hyperbaric oxygen therapy (HBOT) has recently been considered an adequate therapeutic alternative in patients with chronic, non-healing or difficult wounds [21,22]. High oxygen pressure of short duration with atmospheric air is employed in HBOT to obtain tissue hyper-oxygenation. The determination of the dosage is related to O2 toxicity of the brain cells and the number of treatment sessions will envision the clinical profile of each patient as well as the type of disorder. HBOT improves wound healing in different ways [21] with an anti-inflammatory action, down-regulation of cell adhesion molecules and a reduced impact on endothelial leukocytes. Moreover, the killing capacity of leukocytes rises with the interruption of anaerobic bacteria and clostridial α toxin production. HBOT has been reported to enhance angiogenesis and vasculogenesis, also inducing vasoconstriction and edema minimization [21]. 

**Figure 1:**
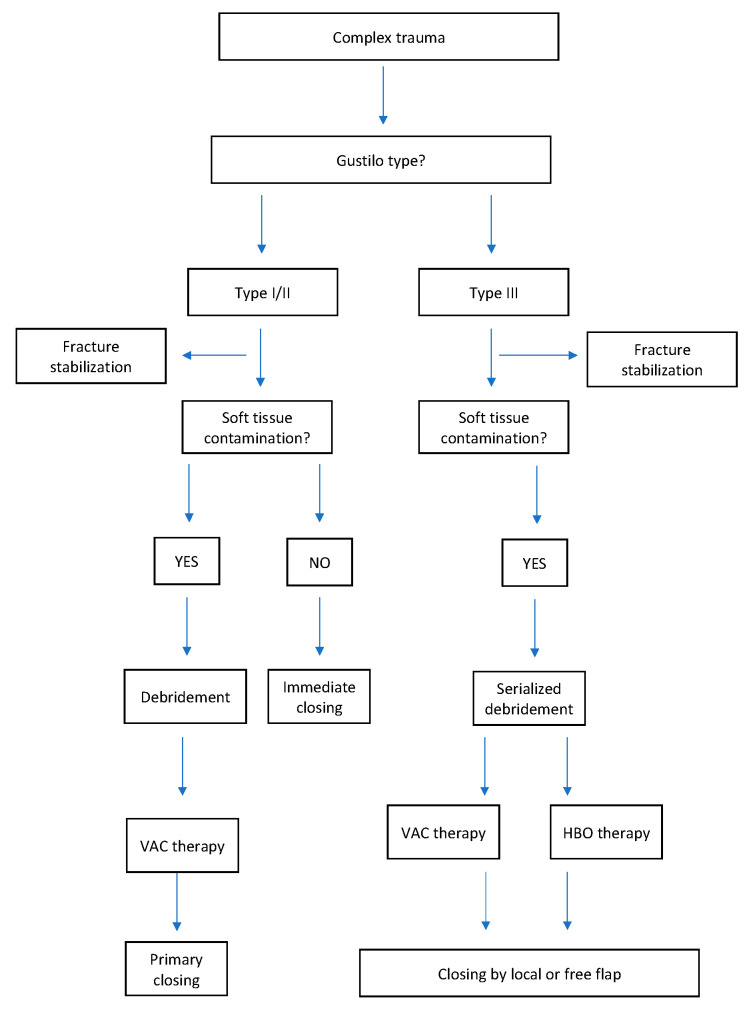
Integrated algorithm before reconstruction in our Hand Reconstructive Microsurgery Department.

HBOT and NPT are considered to be satisfactory approaches to ameliorate tissue oxygenation, to eradicate infection, and alleviate healing. An alternative treatment may be HBOT and NPT in combination with immediate antibiotic therapy together with multiple surgical debridement and physiotherapy. We herein report our integrated protocol (Figure 1) prior to reconstruction based on a 10 year-experience in our reconstructive microsurgery department.

## 2. Materials and Methods

### 2.1. Patient Database

A single-center retrospective study was conducted on all post-traumatic patients affected by complex traumas admitted to our hospital from 2006 to 2018. The study was authorized by the Ethics Committee of Marche Region (CERM) on October 2019 (Project identification code: VAC-OTI_1) (OR566-17). All administered procedures involving human participants were compliant with the ethical standards of the institutional and national research committee and the 1964 Helsinki declaration and later amendments or comparable ethical standards.

One hundred and seventy-nine patients (179) presented with complex upper limb trauma with significant soft tissue injury of the upper limb and numerous degrees of fractures. The Gustilo Anderson classification system for open fractures was employed to identify the fractures [23]. Our inclusion criteria comprised patients with Grade II and Grade III affected by strong contamination, severe soft tissue damage and bone injury. Grade I and a majority of Grade II injuries were excluded due to closed wounds following debridement during primary surgery. Data collection was taken from inpatient and outpatient medical records of our department extracting data including injury dynamics, patient demographics, vascular and neural injury, segmental bone loss, type of fracture, microorganism infection, complications and final functional outcomes. Treatment was defined as (I) wound debridement, (II) application of NPT, and (III) use of HBOT, (IV) administration of oral or intravenous antibiotics, (V) immediate physiotherapy, (VI) tissue reconstruction. The patients were appointed to receive the combined Medical Support Methods and Microsurgery protocol based on such criteria as (I) severe crush limb injury with large tissue loss; (II) massive necrosis areas following 2–3 days of injury; (III) deep post-traumatic ulcers with exposure of joint, bone and tendon structures; (IV) wide and deep wounds affected by exogeneous material; (V) large dystrophic tissue surrounding the ulcer with risk of necrosis and ulceration. 

### 2.2. Treatment

We conducted the surgical treatment as per protocol performing copious irrigation, fasciotomy (if required) and extensive debridement, proceeding to internal fixation with or without external support. We treated arterial lacerations with venous interposition grafts and nerve lacerations were treated conducting nerve repair or nerve reconstruction by means of tubulization or grafting. The precise injury zone is often wider than expected, and a complete single-step debridement is difficult to achieve, hence a multi-stage procedure is needed. 

Perioperative antibiotic coverage was given to the patients as per institutional protocol. Our infectious disease specialists are responsible for antibiotic administration employing protocols in accordance with the type of trauma which are classified into “clean”, “unclean” and “contaminated”. “Clean” traumas are defined as domestic occurrences, “unclean” as working incidents and “contaminated” as high energy traumas from street or rural environments. 

The first group was treated with cefazolin 2 g/day, and the second group was treated with ceftriaxone 2 g/day. The contaminated wounds were treated with meropenem 1 g × 3/day and teicoplanin 400 mg × 2 for the first day then 400 mg/day. Empiric therapy replaced the specific treatment with respect to microbiological cultures from intraoperative wound specimens. These antibiotics were continued post-operatively. In the case of an established yeast infection, targeted pharmacological treatment was duly administered.

All patients without exposure of major vascular or nervous structures, underwent NPT of 125 mmHg according to institutional protocol. NPT will provide a humid environment, reduced edema, greater local blood flow and will enhance angiogenesis, produce tissue granulation, stimulate cell proliferation, decrease the dimension and complexity of the wound, remove soluble healing inhibitors from the wound and diminish bacterial load. The intensity of the negative pressure (ranging from 40 to 150 mmHg), the administration of intermittent or continuous pressure, and the filler material to cover the wound have all been widely discussed in literature. We used the vacuum-assisted closure therapy system (KCL Inc, Verona, Italy) to deliver NPT at a continuous pressure of 125 mmHg and performed the interventions under sterile conditions in an operating theatre. Dressings were changed every 4–5 days at bedside or under sterile conditions with swabs collected for aerobic and anaerobic culture. Once the wound had sufficient granulation tissue and was devoid of infection signs, the site of trauma was reconstructed, by means of a dermal substitute with secondary skin grafts or by means of free or local flaps following the simultaneous removal of the aforementioned granulation tissue not presenting valid tissue for functional recovery of the limb. Serial irrigation and debridement continued for at least 10–15 days if the wounds were not ready for closure or coverage.

The patients, with more complicated trauma or injury, underwent, as per our institutional protocol, one cycle (10 sessions) of HBOT (2.5 bars, 100% oxygen, 120 min with air breaks) within 48 h of surgery. HBOT is based on the rise of dissolved O2 level up to 15 times in areas not presenting blood capillaries. The healing process is O_2_-dependent by its action on macrophages and fibroblasts. The O_2_ activity is less visible but equally important as NPT, maintaining tissue vitality in the hypoxic phase as well as counteracting bacterial growth due to the bactericide effect with a direct oxidative reaction and indirect macrophage action. 

We marked the areas containing viable mangled skin, excised the necrotic skin, then performed immediate or delayed soft-tissue coverage in relation to the overlying granulation tissue and any existing delayed infection.

As soon as the patient felt comfortable, he/she was able to freely move his/her arm supervised by the physiotherapist. The aim of a reconstructive upper limb treatment is indeed to restore function. Trauma, inflammation and surgical treatment cause scar tissue with tendon and joint implication extending to structures that are not directly involved in trauma, thus requiring prompt intervention with physical therapy to avoid complications from delayed treatment. Our therapists were committed to conducting daily manual therapy and the packaging of splint custom tailor-made for each patient.

## 3. Results

A total of 894 post traumatic surgeries (upper and lower limb) were performed from 2006 to 2018 in our Hospital. Of these, 179 (20%) patients were diagnosed with a complex trauma of the upper limb, with complicated infection, subject to therapy and are reported herein—129 (72%) males and 50 (28%) females with a mean age of 48 years (range: 36–62 years). Seventy nine patients presented with road injuries (44%)—motor vehicle versus pedestrian (4%), motorcycle collisions (18%), and motor vehicle versus vehicle or object (22%)— while 82 patients were victims of work accidents (46%). The most frequent post traumatic tissue loss injuries included tendons and muscles (43%), and bones (42%). Five patients presented with head injury, three patients with a clavicle fracture, five patients with lung insult and rib fracture and twenty patients with a lower limb fracture (Table 1). No significant differences in age, smoking status, diabetes, hypertension, immune status or mechanism of injury were observed between patients who were subjected to NPT or HBOT and those not subjected to the respective management.

Debridement of the wounds was urgently performed in all (100%) patients and biological tissue samples were collected for cultural examination. Wound cultures revealed organisms such as *Staphylococcus aureus* (63%), methicillin *resistant Staphylococcus aureus* (MRSA) (23%), *Pseudomonas Aeruginosas* (7%), *Acinetobacter baumanii* (4%), and others (3%). The presence of MRSA and Baumani was, however, limited to chronic cases from long hospitalizations and/or in intensive unit care.

The following antibiotics were given: meropenem, teicoplanin, vancomycin, cefotaxime, amoxicillin, and daptomycin before flap coverage or on recipient site infection with no significant difference in administration of systemic antibiotics and application of NPT and HBOT. The duration of the antibiotic lasted for the entire hospitalization and for approximately 10 days after hospital discharge. Of 75 patients with bone fractures, the most common fracture classification was Gustilo III (43 patients, 58%) and Gustilo II in 32 patients (42%). The complicated wound infections comprising 42% of the patients were treated with NPT and 58% of the patients with NPT + HBOT. The less complex cases, without exposure of bone structures, did not require the use of HBOT. The mean length of stay was 23 (±2) days, the median stay lasted 24 days, with a range of 21 to 25 days (Table 2).

**Figure 2:**
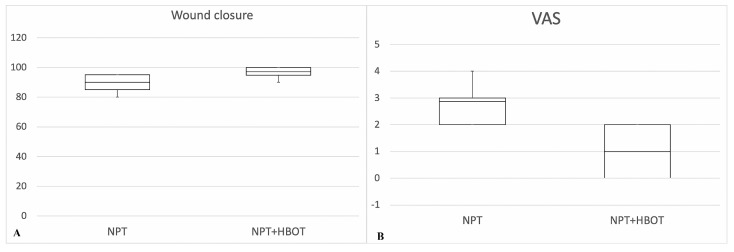
(**A**). Wound closure rate (%) comparison between use of NPT alone and in combination with HBOT. (**B**) Pain assessment (VAS) comparison between use of NPT alone and in combination with hyperbaric oxygen therapy (HBOT). Data analysis is displayed in box and whisker plot (minimum, first quartile, median, third quartile, and maximum) and assessed via a two-tailed *t*-test for comparison.

Survivors completed wound healing within 15 days from the time of diagnosis with a mean duration of 15 days and a median of 15 days in each group. Patients in each group were discharged after 28 (±3) days (range: 25–31 days; median: 28 days) from admission. The duration of HBOT lasted an average of 10 (±2) days (range: 8–10 days), and the average number of HBOT cycles of therapy was 10 (±2). The average number of interventions from the first debridement to the first free flap reconstruction, or local flap coverage or skin graft was three (a range of one to six times). The most frequently performed tissue transfer was free or reverse radial forearm flap, free anterolateral thigh flap, free or pedunculated latissimus dorsi flap, free fibula flap and free gracilis flap. None of the patients in the NPT + HBOT following reconstructive flap treatment group were affected by deep infection. The mean wound closure was 90% (±12%) in the NPT patients, revealing a considerably lower value (*p* < 0.05) than in the NPT + HBOT patients (97% ± 13%) (Figure 2A) and the mean VAS for postoperative pain was 2.7 (±1) in the NPT patients and 1 (±1) in the NPT + HBOT patients (Figure 2B).

### 3.1. Case Study #1

A 66-year-old female patient presented with a severe avulsion and crush injury of the left hand subsequent to a car accident. The injury had provoked laceration of the extensor tendon for I-II and III finger with loss of tissue and V metacarpal bone fracture. Initially, the wound appeared to be 4 × 5 cm in dimensions. After initial debridement and metacarpal bone fixation, the patient was subjected to a combined therapy with NPT and ten HBOT sessions. 

**Figure 3:**
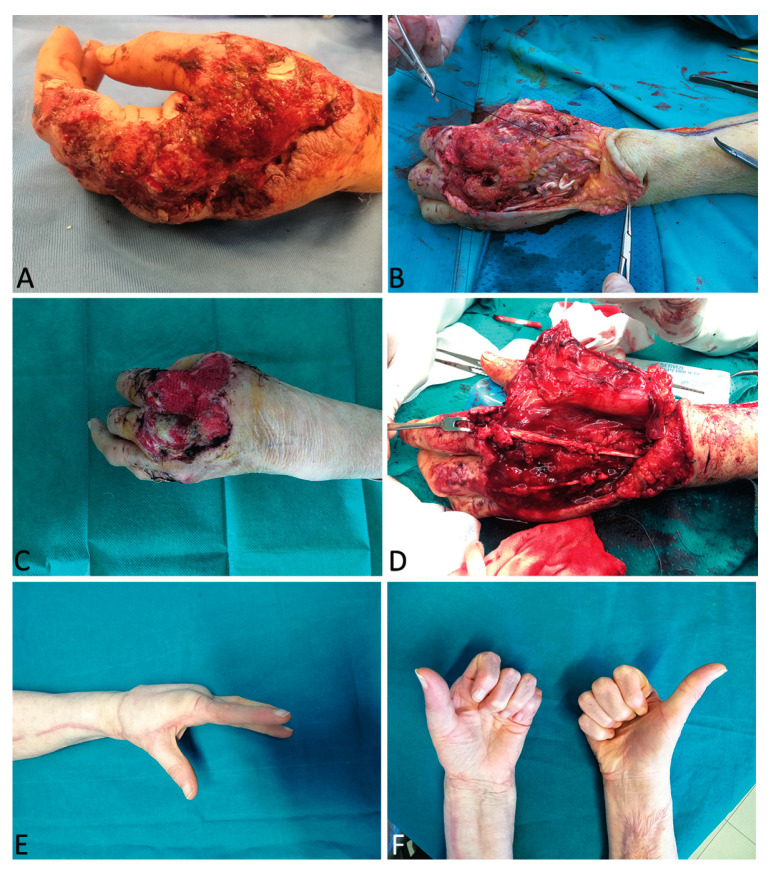
A 66-year-old female patient presented with a severe avulsion and crush injury of the left hand subsequent to a car accident (**A**). After initial debridement and metacarpal bone fixation (**B**), the patient was subjected to a combined therapy with negative pressure therapy (NPT) and ten HBOT sessions (**C**). Reconstruction was performed with a contralateral radial forearm composite free flap for wound coverage and restoration of finger extension (**D**,**E**,**F**).

Further debridement for extreme contamination and progressive enlargement of the necrotic areas was required. Antibiotic protocol for contaminated trauma was applied with meropenem and teicoplanin. This therapeutic procedure continued for 20 days, during which the patient was treated by a hand therapist to maintain joint motion. Three weeks after trauma with a stable wound (increased up to 9 × 6 cm) and no evidence of further bacterial load, reconstruction was performed using a contralateral radial forearm composite free flap with palmaris longus and a strip of flexor radialis carpis and brachioradialis tendon to cover the wound and restore finger extension. Hand therapy was not discontinued with the exception of the first 5 days following free flap treatment. The patient healed uneventfully. At one year follow-up, we observed no tissue dystrophy and the covering tissue appeared trophic and well-vascularized. The total range of motion of the left fingers was sufficiently comparable to the healthy side and the patient returned to her normal daily activities (Figure 3).

### 3.2. Case Study #2

**Figure 4:**
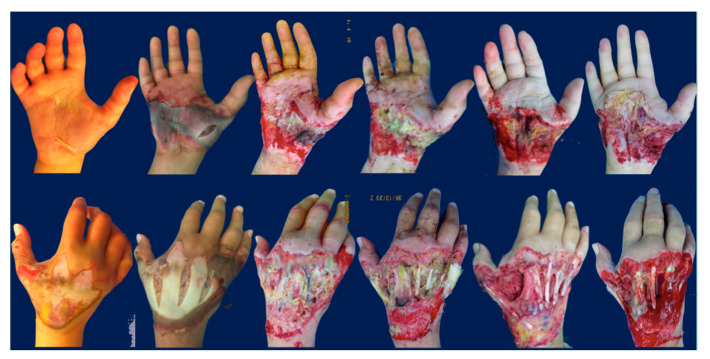
A 22-year-old female patient was treated for a severe injury of the right hand following a work trauma under a hot press. This lesion had caused a compartmental syndrome with extensive necrosis of superficial and deep tissues and composite multiple metacarpal bone fractures. After 4 weeks and 6 different debridements for progressive enlargement of the necrotic area, the exact extent of the trauma was visible and involved palm and dorsum circumferentially.

**Figure 5:**
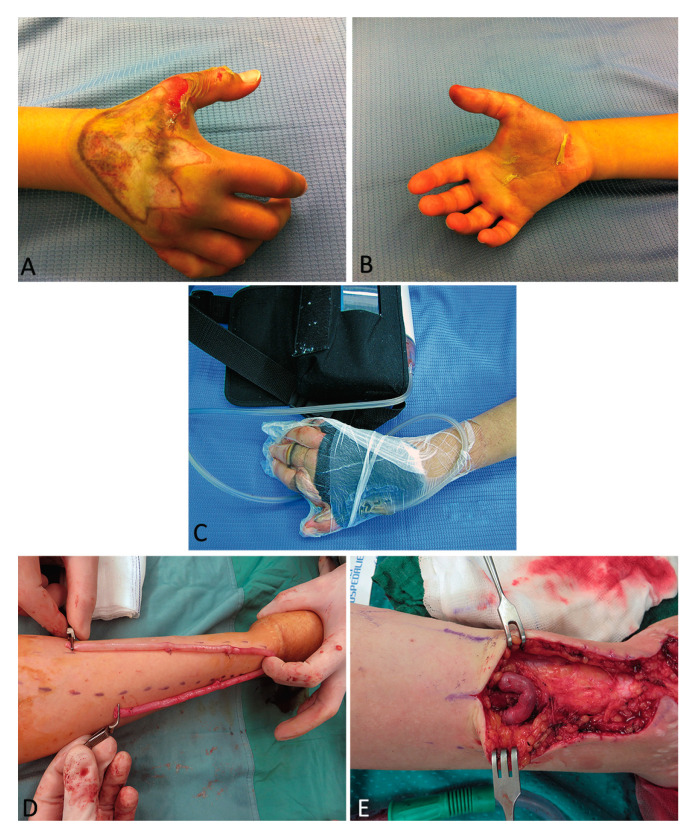
A 22-year-old female patient was treated for a severe injury of the right hand following a work trauma under a hot press (**A**,**B**). Combined therapy with NPT and 10 + 5 HBOT sessions was associated (**C**). Soft tissue reconstruction was performed with an anterolateral thigh flap. For the second-step bone reconstruction, we performed a vessel loop with saphena to provide a new vascular pedicle outside the injured zone (**D**,**E**).

A 22-year-old female patient was admitted and treated for severe injury of the right hand following a work trauma under a hot press. This lesion had caused compartmental syndrome with extensive necrosis of the superficial and deep tissues as well as composite multiple metacarpal bone fractures. Antibiotic protocol for “unclean” trauma was applied using ceftriaxone. Multiple surgical sessions were performed to clean dead tissue. Combined therapy with NPT and 10 + 5 HBOT sessions was performed. After 4 weeks and six different debridements for progressive enlargement of the necrotic area, the exact extent of the trauma was visible and involved palm and dorsum circumferentially (Figure 4 and Figure 5A–C). 

The patient presented with tendon and neurovascular pedicle sparing necrosis involving all layers up to the fractured metacarpal bone. Soft tissue reconstruction was performed with an anterolateral thigh flap subsequently defatted. In the following months, X-rays revealed no bone healing process, and in the medium-term a type of bone reabsorption was evident. Necrosis depth was more significant than expected, thus bone replacement in a mono-vessel limb was required, precisely the ulna, since the radial pedicle, previously interrupted by the trauma at wrist level, was used in a termino-terminal fashion to vascularize the ALT flap. We planned a two-step bone reconstruction. Firstly, we removed the necrotic third and fourth metacarpal bones, inserting a bone spacer with an antibiotic and simultaneously performed a vessel loop with saphena to provide a new vascular pedicle outside the injured zone (Figure 5D,E).

**Figure 6:**
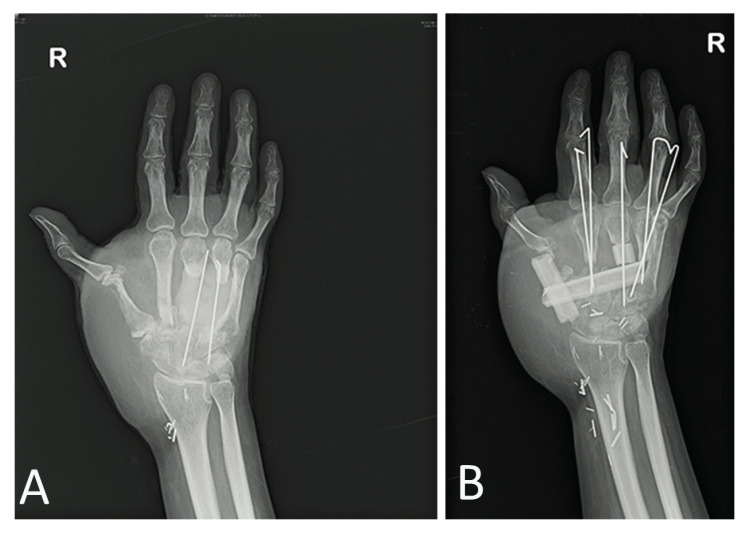
After 1 month, we performed a fibula flap with definitive 1st to 4th metacarpal bone replacement (**B**) following reabsorption observed during the operation (**A**).

After one month, we performed a fibula flap with definitive first to fourth metacarpal bone replacement as a consequence of further reabsorption observed during the operation (Figure 6A,B).

## 4. Discussion

A complex wound is commonly a result of a high energy traumatic event with lengthy and complex treatment procedures. The injury zone is a dynamic entity and is rarely visible in its authentic state during emergency intervention [24,25,26,27]. The type of trauma is to be initially identified together with analysis and expectations of the mutilated limb. As regards the upper limb, the prehensile function of the hand is to be specifically preserved as the position of the thumb, and the ability to use the hand during daily activities. In the case of an unsuccessful achievement of daily functions, the traumatized hand will be an appendage and amputation should be considered. This surgical time is complicated both for the evolution of the lesion and for any underestimated adverse events. In case #2, we report our experience and the necessity to perform six debridements to disclose the precise extension of the soft tissue wound. The traumatic heating mechanism had progressively extended the area of the lesion, which reveals the possibility of wasting reconstruction due to underestimation of the injury with consequential wound dehiscence, abscess formation with local extension of scar tissue as well as systemic risk. Due to the dynamic nature of the wound, the “peripheral gray area” is to be rendered visible to allow for diminishment in wound size, conceivable via surgical debridement used previously, removing all necrotic tissue which would provide additional inflammatory events. Hopf et al. [28] and Saxena and colleagues [29] emphasized the importance of wound debridement to commence the healing process and in the light of this information we herein, adopted the same principle, removing necrotic tissue before therapy to provide optimal conditions for the healing process and to assess the therapeutic options to adopt—only NPT or NPT in combination with HBOT.

Both NPT and HBOT improve local flow, diminishing local edema and driving more perfusion into a targeted area [30]. Both NPT and HBOT stimulate neoangiogenesis perceivable with improved quality granulation tissue in the wounds [31]. Moreover, both NPT and HBOT are able to produce a synergistic effect on infection control and to eliminate local tissue infections. Although both may possess anti-infective properties, infection and necrosis should be regulated with surgery and antibiotics. The clinical outcomes of NPT are widely presented in the literature [32]; however, salvage of the so-called “peripheral gray area” has been difficult to achieve with NPT. HBOT has also recently been approved to treat patients with chronic, non-healing or complicated wounds [33]. High pressure oxygen at a short duration and atmospheric air are considered to hyper-oxygenate the tissues, acting directly on the tissues and indirectly on the cells [34] revealing anti-anti-inflammatory potential and down-regulation of the molecules in cell adhesion and reduction in leukocyte endothelial influence. Therefore, both NPT and HBOT possess prerequisites for safety usage. We suggest using this combined approach (NPT + HBOT) in cases where “damage control” is required and specifically when a temporary external fixation of the fractures is performed with wounds left open and controlled by vacuum dressing. This approach, on the one hand, lengthens the time of the definitive orthopedic and plastic-reconstructive approach, but on the other hand, reduces the systemic complications of trauma such as crush injury and compartment syndrome. This procedure is applicable if the functional preservation of the limb is the complex management objective. Recent investigations revealed advances in orthopedic, vascular and plastic surgical management of limb recovery to prevent a widely debated early amputation as opposed to limb salvage [35]. The management of severe open fractures has also been extensively addressed and the issue of early soft tissue coverage with free tissue transfer [36,37]. These procedures have indeed yielded satisfactory results and extremely low failure rates, but the techniques are remarkably demanding and often lead to complications of the donor site. Studies on lower limb injuries have recently proposed successful options using appropriate surgical debridement and vacuum dressings, thus reducing the possibility to use free soft tissue transfer yielding equally optimal outcomes [38,39]. The possibility for HBOT to diminish edema and avoid necrosis and infection will enable a less complicated soft tissue closure with sufficient opportunity to plan relevant orthopedic interventions and plastic surgery. The objective of our combined study is to curb ischemic events, to preserve any partially viable tissue and define lesion areas. Mangled hand lesions present with an initial un-retrievable tissue deterioration and urgent need of debridement, but HBOT may support and optimize jeopardized tissue and regenerate perfusion. Indeed, HBOT may offer a more efficient therapeutic alternative as the extent of the trauma increases. We initiated HBOT treatment in our study within 24 h following revascularization or stabilization of the mangled upper arm displaying clear wound demarcation and reduction of infectious complications.

Mangled upper arm injuries are commonly prone to receive further surgical interventions to rectify stiffness, contractures, adhesions and nonunion, in addition to ensuring functional restoration in chronic stages. Physiotherapy and early rehabilitation may avoid regression. Immobility facilitates adhesions and edema formation creating scar tissue with joint and tendon involvement. Evolution of Case #1 was less complicated as regards tissue involvement and trauma mechanism due to crush-avulsion, sparing deep structures. The aim was to restore extension of the fingers, accessible via a tendon reconstruction acting on a mobile joint. Physiotherapy was thus administered since day 4 following the trauma and continued for the entire hospital stay. This allowed for a one-step tendinocutaneous radial forearm free flap restoring the first, second and third finger extensions.

Our results on the mangled arm permit the composite strategy, despite long hospitalization time. Godina suggested that early microsurgical reconstruction within 72 h of trauma would improve flap endurance, diminish infection and abbreviate hospitalization time [10]. This notion has nevertheless been contested by numerous authors [40,41,42], with some investigators reporting that reconstruction may occur up to two weeks from trauma, claiming furthermore that flap endurance was unrelated to time interval. We revealed a reduced necessity to use free tissue transfers for Gustilo III traumas and observed that NPT and HBOT improved granulation tissue formation and restored blood flow to the wound bed.

## 5. Conclusions

The therapies NPT and HBOT have indeed allowed for satisfactory wound coverage, extending the time-lapse until upper-limb reconstruction is required. In the clinical scenario herein, attentive surgical planning was required prior to treating complex traumas to optimize time and methods and with the objective to restore the necessary functions of the limbs. The use of ancillary therapies (NPT, HBOT, ATB) is fundamental together with an adequate organization of the various phases in the reconstructive process. The gold standard for the treatment of complex tissue has yet to be defined. The use of this integrated protocol is made both after the first debridement in the emergency room and after 4 days when we have the response of the bacteriological tests. The decision to perform both NPT and HBOT is whether we have uncovered exposed bone following the first debridement and whether we have multiresistant or complicated bacteria in Gustilo III severity patients. This approach provides fundamental wound coverage but most importantly offers satisfactory functional results. We should outline that this study had some limitations related to the fact it is retrospective, and the sample, although numerous, is distributed over a very long period. Moreover, we can affirm that there could be a lack of randomization because we could not include a control group. Our report reveals favorable outcomes in the treatment of complex mangled upper limb trauma using a multi-mode technique combining NPT and HBOT with an adequate management of antibiotics.

## Figures and Tables

**Table 1:** Patient demographics and mechanism of injury.

	Number of Patients	Percentage
**n (patients)**	179	
**Gender**		
Male	129	72%
Female	50	28%
**Smokers**	20	11%
**Medical disease**		
Hypertension	8	4%
Diabetes mellitus	14	8%
Immunosuppressed	2	1%
**Mechanism injury**		
Auto versus pedestrian	7	4%
Motor vehicle accidents	39	22%
Motorcycle collisions	33	18%
Work crushing	77	43%
Work blast	5	3%
Gunshot wound	8	5%
Animal attacks	10	6%
**Post traumatic loss injuries**		
Skin	28	15%
Tendon and muscles	76	43%
Bone	75	42%
**Associated injuries**		
Head injury	5	3%
Clavicle fracture	3	2%
Lung insult and Rib fracture	5	3%
Lower limb fracture	20	11%

**Table 2:** Injury characteristics.

	Number of Patients	Percentage
**n (patients)**	179	
Skin loss	28	15%
Soft tissue loss only	76	43%
Bone fractures	75	42%
Debridement	179	100%
**Microorganism infection (%)**		
Staphylococcus aureus		63%
Staphylococcus aureus MRSA		23%
Pseudomonas Aeruginosas		7%
Acinetobacter baumanii		4%
Others		3%
**Systemic antibiotics**	179	100%
Fracture severity		
Gustilo II	32	42%
Gustilo III	43	58%
**Location of fracture**		
Medical Support Methods (%)		
NPT	32	42%
NPT + HBOT	43	58%
